# Medical History from the Earliest Times

**Published:** 1893-05-13

**Authors:** E. T. Withington


### Medical History from the Earliest Times.

By E. T. Withington, M.A., M.B. Oxon.

### LIV.—VAN HELMONT.

John Baptist van Helmont (1577-1644) is almost as striking a figure in medical history as Paracelsus, whom he resembles in many respects, though he surpassed him both in genius and learning. His character is so closely connected with his doctrines, that it will be necessary to give a few biographical details. The youngest son of a noble Flemish family, Van Helmont lost his father in childhood, and received a pious and strictly Catholic education from his mother. He then went through the University course at Louvain, but refused the degree of M.A., declaring that so far from being "Master" of the seven liberal "Arts," he was not even a disciple in one, and that his knowledge, like Adam's, had served only to show him his nakedness. After dabbling in magic, he betook himself to the study of ethics as the only worthy form of knowledge, and read especially the works of Seneca, Epictetus, Thomas à Kempis, and John Tauler, a mixture of Stoic philosophy and Christian mysticism, which did not fail to produce a moral nightmare. He dreamt he saw himself like a huge empty bladder reaching from earth to heaven, but above him, instead of heaven, was a coffin, and below him the blackness of the abyss. So he saw that Stoicism is but a form of pride, and man, without the inspiration of God, a thing of nought and a vain shadow. A rich living was offered him if he took orders, but he was frightened by the saying of St. Bernard, that he would live from the sins of the people. Then he thought of law, but soon found that its ordinances were often far removed from truth and justice, and how should he guide and restrain others who found it hard enough to govern and direct himself? So he turned to Dioscorides and Matthioli to study the goodness of God in the virtues of herbs, and this led him to medicine. Here, he thought, was a science in which a student bent upon relieving the miseries of his fellows could not fail to receive Divine assistance, and the great teachers of which must be men inspired of God, even as were Bezaleel and Aholiab, the builders of the Tabernacle. He carefully studied all the approved medical authors, taking notes of everything that seemed valuable; then he read the notes and was overwhelmed with disappointment and despair. "I said in my heart, 'O merciful God, how long wilt Thou be angry with man, that Thou hast not revealed one truth to Thy students in healing? Is this Moloch sacrifice pleasing to Thee, and wilt Thou that the lives of the poor, of widows, and of children be continually offered up to Thee in miserable torments of incurable diseases or through the carelessness and ignorance of physicians?' Then I fell on my face and cried, 'Lord have mercy! pardon, pardon me, O Lord, if love of my neighbour has led me beyond bound, for Thou art the root of all goodness, Thou knowest my sighing, and that I confess I am empty, ignorant, poor, and naked, and have nothing, am nothing, and can do nothing.'" But he was encouraged by a dream to persevere in his work, and having taken the degree of M.D. at Louvain, set himself no less a task than "to overthrow the entire philosophy of the ancients and establish a new science of Nature." The existing medicine, he says, is based on that of the pagan Greeks, and considers only the outside of things, but Christian science like Christian morality must begin from within.

To Van Helmont all Nature is alive; there is no such thing as dead matter, but in animals this material life, as we may term it, assumes an almost personal form, which he calls "Archeus," a word already used, though more vaguely, by Paracelsus and Basil Valentine. Every bodily structure has its local "Archeus insitus," and the whole organism is governed and directed by an "Archeus influus" who builds up the body and supports its activity, working always according to a fixed plan, or "seminal idea" impressed by the Creator. This Archeus resides in the stomach, and is closely connected with the sensitive and rational soul, the two together forming a sort of husk or shell for the higher intuitive or intellectual spirit. For Van Helmont distinguishes between intellect and reason, and declares that the latter is an inferior quality, acquired by, and necessary to man since the Fall, but leading nearly as often to error as to truth. The heathen Aristotle in his blindness bowed down to reason, and set it up as an idol in the form of Logic, a vain science, which deals only with externals and definitions. One definition, indeed, is important in medicine—that of disease, for it is not a mere matter of words, but affects the lives of men; and here the pagans and their Christian disciples have gone hopelessly astray in spite of their logic. For they call disease "a lesion of structure or function," and declare that its cause is a change in the fluids or humours, the disease itself being in the solids, while the spirit or "pneuma" directs the symptoms. The reverse is the truth, for disease affects the life, and must, therefore, have its seat in the life, or Archeus, through whose action it produces changes in the solids and fluids. How can an organic lesion be a disease, for it persists in dead bodies, and the dead can have no diseases? "Whatever produces healthy actions in the sound, the same causes vitiated actions in diseases." (Quidquid in sanis edit actiones sanas, idipsum in morbis edit actiones vitiatas.) Disease is a morbid idea conceived by the Archeus, either through his own infirmity, or from the action of some harmful agent, which causes him to deviate from his normal course and act in another way, but always after some fixed and specific plan. There are thus two great divisions —(1) innate diseases, under which head he classes inherited and latent affections, such as epilepsy, and (2) those due to external agencies, among which the results of witchcraft, "injecta a sagis" play a large part.

He applies these doctrines to special instances with much ingenuity. Fevers, say the Galenists, are either simple, and due to preternatural heat originating in the heart, or putrid, caused by a corruption of the humours, especially the blood; and they treat them by bleeding, purgation, and cooling medicines. This, according to Helmont, is false, for heat is merely a symptom, and the blood never putrefies during life. Fever is the effort of the chief Archeus to get rid of some irritant, just as local inflammation is the reaction of the local Archeus to some injury. The heat is caused by the anger of the Archeus, who tries to shake off his enemy by calling up cold and hot fits, a "blas frigoris" and "blas caloris," and finally a "blas alterativum," or fœtid sweat. The intermittencies are due to his taking breath for another effort, and the therapeutic indication is to assist him by giving support, especially by wine, and to imitate his action by the use of diaphoretics. Bleeding is Helmont's special abomination, for nothing is more calculated to weaken and irritate the Archeus. "I see a blood-stained Moloch presiding at the councils of physicians. Repent, repent, therefore, my brethren, for there cometh a terrible day upon the world at the sound of the trumpet, when every man shall give an account of his deeds." Catarrhs, according to the Humoralists, are caused by phlegm rising to the brain, where it is condensed as by a cold dish cover, and flows down into the nose, eyes, lungs, joints, and muscles, giving rise to coryza, bronchitis, and different kinds of rheumatism, diseases to be treated mainly by purgatives, demulcents, and "hot" medicines. Helmont calls these theories absurdities, "catarrhi deliramenta." Phlegm is secreted by the local Archei, or "guardians," as he here terms them, to protect the tissue from irritants; but if the irritation continues the guardian gets reckless, he becomes a "custos errans," and secretes mucus of bad quality and excessive quantity, so as to block up important passages, which the presiding Archeus has to reopen by setting up sneezing and coughing. The proper treatment is obvious. In slight cases nothing more is required than to remove the cause of irritation; in severe coryza he recommends a sternutatory or snuff of powdered hellebore and sugar, equal parts, and a reduction of diet, especially in fluids, so as to cut off the errant guardian's supplies. Sulphur is sometimes useful in bronchitis, and when the Archeus overdoes the coughing he may be restrained by the use of narcotics.

Divested of its fantastic language, we must admit that there is much valuable truth in the above, and Van Helmont rendered additional services to medicine by his chemical researches, which resulted in the discovery of carbon dioxide, and in the proof that an acid takes part in gastric digestion. He was directed to chemistry by his early admiration for Paracelsus, but soon became dissatisfied with his master, and his refutation of the latter's most characteristic theories is not the least important part of his work. The doctrine of signatures, says Helmont, is false, and rests on the pagan delusion of the microcosm. "I believe that God reveals remedies to whom He will by special grace, not 'per signa Naturæ,' forman is not the image of Nature, but of God." He similarly attacks the Paracelsic doctrine of "tartar" and of the mystic elements, salt, sulphur, and mercury. But, on the other hand, he strongly upheld the virtue of the sympathetic ointment, and his works abound in absurdities surpassing even those of Paracelsus. We have only space for one example. Dropsy is, he says, not due to an organic lesion of the liver, but to the anger of the renal Archeus, who has lost his temper, and refuses to work. One way of reducing him to order is to terrify him, and this may be done by tying a snake round the patient's waist and applying live toads to the region of the kidneys.

